# Rectal Surveillance for Carbapenem-Resistant *Klebsiella pneumoniae*: Prevalence and Clinical Relevance in a Multidisciplinary Tertiary-Care Hospital

**DOI:** 10.3390/antibiotics15080764

**Published:** 2026-08-10

**Authors:** Cristiana Ana-Maria Olguța Penea, Violeta Melinte, Elena Crețu, Tiberiu Holban, Adelina Maria Radu, Cristina Maria Vacaroiu, Claudia Simona Cambrea, Valeriu Gheorghiță

**Affiliations:** 1 “Agrippa Ionescu” Clinical Emergency Hospital, 011356 Bucharest, Romaniavaleriu.gheorghita@umfcd.ro (V.G.); 2 Department of Infectious Diseases, “Carol Davila” University of Medicine and Pharmacy, 050474 Bucharest, Romania; 3 Faculty of Medicine, “Ovidius” University of Constanța, 900470 Constanța, Romania

**Keywords:** *Klebsiella pneumoniae*, carbapenem resistance, gastrointestinal carriage, rectal surveillance, healthcare exposure, antimicrobial susceptibility testing, infection prevention, antimicrobial stewardship

## Abstract

**Background/Objectives:** Carbapenem-resistant *Klebsiella pneumoniae* (CRKP) is a high-priority healthcare-associated pathogen. This study aimed to estimate rectal CRKP carriage and characterize same-patient clinical-site CRKP detection. **Methods:** We conducted a single-center retrospective microbiological surveillance study in a Romanian tertiary-care hospital between 1 January 2025 and 1 April 2026. Patient-level prevalence used all 3123 screened patients, with each patient counted once. CRKP recovery from rectal screening specimens was classified as gastrointestinal carriage; non-rectal recovery was reported as clinical-site detection and not as confirmed infection because clinical adjudication was not systematic. **Results:** Among 3123 screened patients, 712 (22.8%) had a positive rectal screen, 294 (9.4%) had resistant rectal *K. pneumoniae* carriage, and 168 (5.4%) had rectal CRKP carriage. Of these 168 carriers, 83 (49.4%) also had a retained non-rectal CRKP isolate; rectal recovery occurred first in 45, non-rectal recovery first in 36, and collection timestamps were identical in 2. Healthcare exposures, prior carbapenem-resistant Enterobacterales carriage, and admission-relative timing were analyzed descriptively using the full 168-patient denominator, with unavailable values retained as unknown. Invasive-device, intensive-care, recent hospitalization or readmission, and recent antimicrobial exposures were frequently documented. Admission-relative timing identified early detections, probable within-admission screen conversions, hospital-onset detections without a preceding negative screen, and indeterminate cases. **Conclusions:** Rectal CRKP carriage was frequent and commonly associated with same-patient CRKP detection at non-rectal clinical sites, indicating a substantial hospital-associated burden. In critically ill patients with sepsis or septic shock, known CRKP carriage should inform the choice of empirical CRKP-active therapy, with prompt de-escalation once microbiological findings and clinical response allow.

## 1. Introduction

Carbapenem-resistant *Klebsiella pneumoniae* (CRKP) is recognized as a high-priority antimicrobial-resistance threat with major implications for hospital infection prevention and antimicrobial stewardship [1,2,3]. *Klebsiella pneumoniae* is a clinically important Gram-negative pathogen with a marked ability to colonize the gastrointestinal tract and cause healthcare-associated infection [4,5]. In clinical practice, it may be recovered from urine, blood, respiratory specimens, wound or secretion samples, collections, and device-associated specimens. In hospitalized patients, gastrointestinal carriage is clinically relevant because it represents a reservoir for transmission and may precede CRKP recovery from clinical specimens at other anatomical sites [6,7,8,9].

Carbapenem-resistant *K. pneumoniae* raises particular concern in hospital settings, where therapeutic options remain limited and transmission can be difficult to control once colonized or infected patients are identified [10,11,12]. International data show that CRKP dissemination is shaped by high-risk clones, carbapenemase-carrying plasmids, and patient movement across healthcare networks [5,13,14]. The European burden of carbapenem-resistant Enterobacterales remains heterogeneous, with regional differences in resistance burden and carbapenemase distribution [15,16].

This study estimated screened population prevalence of rectal *K. pneumoniae* and CRKP carriage and described same-patient clinical-site detection, collection order, healthcare exposures, prior CRE carriage, and admission-relative timing among all 168 carriers. These analyses were descriptive and did not establish infection, independent risk factors, acquisition source, progression, transmission, or clonal relatedness.

## 2. Materials and Methods

### 2.1. Study Setting, Data Sources, and Analytic Units

This single-center retrospective microbiological surveillance study was performed at “Prof. Dr. Agrippa Ionescu” Clinical Emergency Hospital, Bucharest, Romania. The study period covered 1 January 2025 through 1 April 2026, inclusive. During the study period, rectal surveillance was performed as part of routine institutional infection prevention practice and was implemented for selected cases with risk factors for carriage. Rectal swabs were requested across intensive-care, surgical, and medical wards according to ward-level clinical and infection-control practice. Hematology and oncology were included within the medical-ward category. Patient-level counts were based on the final deduplicated setting classification available in the analytic dataset. Hospital administrative statistics were used to establish the hospital-level denominator. During the study period, the institution recorded 21,122 admission episodes. Because the administrative denominator represented admission episodes rather than deduplicated individuals, the relationship between unique screened patients and hospital activity was interpreted as a screening-to-admission ratio rather than person-level hospital-wide screening coverage. Data were extracted from microbiology laboratory exports. The primary extraction unit was the laboratory record. Organism-specific results were assigned to standardized anatomical site categories. For source-stratified isolate analyses, the earliest chronologically documented isolate was retained for each unique patient identifier, anatomical site category, and microorganism. The exports included diagnostic clinical specimens, rectal surveillance swabs, repeated cultures, and supplemental antimicrobial susceptibility testing procedures. Records corresponding only to antimicrobial susceptibility testing follow-up procedures were excluded when they did not represent a new specimen. When multiple antimicrobial susceptibility segments referred to the same microorganism within the same source record, they were consolidated before deduplication. Repeated isolates of the same microorganism from the same site in the same patient were then excluded.

The screened population comprised all unique patients with at least one rectal surveillance swab, irrespective of result; 6742 swab records were retained only as a workload measure. A patient was classified as rectal screening positive after any positive rectal result and as having rectal *K. pneumoniae* carriage after any qualifying rectal isolate during the study period. Rectal CRKP carriage required at least one qualifying rectal CRKP isolate, irrespective of earlier non-CRKP results. Patients with resistant rectal *K. pneumoniae* but no rectal CRKP isolate were classified as non-CRKP resistant *K. pneumoniae* carriers. The earliest rectal CRKP isolate was used only as the chronology index; source-stratified analyses retained the earliest isolate per patient, anatomical site, and microorganism.

### 2.2. Microbiological Definitions

The analysis included *K. pneumoniae* recovered from rectal screening and non-rectal clinical specimens. Resistance-focused rectal screening targeted ESBL-producing and carbapenem-resistant Enterobacterales and did not quantify susceptible intestinal *K. pneumoniae*. CRKP recovery from rectal screening specimens was classified as gastrointestinal carriage. Because infection status was not systematically adjudicated, recovery from non-rectal clinical specimens was reported as clinical-site detection and not as confirmed infection.

At the isolate level, CRKP was defined phenotypically as a retained *K. pneumoniae* isolate resistant to at least one of ertapenem, imipenem, or meropenem. All isolates were cultured under aerobic conditions at 37 °C using routinely employed culture media. Conventional media included Columbia agar supplemented with 5% sheep blood and MacConkey agar (bioMérieux, Marcy-l’Étoile, France). Selective chromogenic media, including chromID^®^ ESBL agar and chromID^®^ CARBA SMART agar (bioMérieux, Marcy-l’Étoile, France), were used when indicated according to routine laboratory protocols. Bacterial identification and routine antimicrobial susceptibility testing were performed using the VITEK^®^ 2 XL automated system with VITEK^®^ 2 identification and antimicrobial susceptibility testing (AST) cards (bioMérieux, Marcy-l’Étoile, France). Antimicrobial susceptibility results were interpreted according to the European Committee on Antimicrobial Susceptibility Testing (EUCAST) criteria applicable at the time of testing [17,18]. CRKP classification relied on VITEK^®^ 2 AST results; confirmatory carbapenem-resistance testing was not uniformly documented in the retrospective exports. Colistin minimum inhibitory concentrations were determined by broth microdilution [19] and interpreted using the EUCAST breakpoints applicable at the time of testing [17,18]. Quality control procedures could not be verified by the retrospective exports.

Clinical-site CRKP detection was defined as recovery of CRKP from a non-rectal clinical specimen, including urine, blood, respiratory specimens, secretions, wounds, collections, mucosal samples, and device- or drain-associated specimens. Same-patient overlap required rectal CRKP carriage and at least one clinical-site CRKP isolate; it indicates microbiological coexistence but does not establish infection or progression from carriage. The analysis focused on microbiological burden, screened population prevalence, anatomical distribution, chronology, and phenotypic resistance; clinical outcomes were not evaluated.

A secondary descriptive analysis summarized healthcare exposures among all 168 patients with rectal CRKP carriage after linkage by unique patient identifier. Variables were intensive-care exposure, hospitalization or readmission within the preceding 30 days, antimicrobial exposure within 3 months, and six invasive-device fields. A documented “yes” classified exposure as present, an explicit “no” as absent, and unavailable or indeterminate information as unknown. Composite device and recent-care variables used the same hierarchy. The database field for prior CRE carriage within 3 months was summarized similarly; because the underlying microbiological event date was not uniformly retained, it was interpreted as recorded history rather than acquisition source. All 168 patients remained in the denominator.

### 2.3. Statistical Analysis

Descriptive statistics summarized workload, deduplicated isolates, and patient-level denominators. Patient-level prevalence used all 3123 unique screened patients; 6742 swab records were not treated as independent isolates. CRKP carriage considered any qualifying rectal CRKP isolate during the study period. Screening-to-admission ratios were expressed per 100 admission episodes, and subgroup and setting distributions as counts and percentages.

Categorical variables were reported as counts and percentages. Phenotypic resistance comparisons between specimen-source categories used drug-specific denominators because antimicrobial susceptibility testing was not uniform across all isolates. Fisher’s exact test was used for 2 × 2 comparisons when expected cell counts were small. Pearson’s chi-square test was used when assumptions for chi-square testing were met. Holm–Bonferroni correction was applied to multiple antimicrobial-resistance comparisons [20].

Chronology was assessed using specimen collection date and time after patient–site–organism deduplication. First-event chronology compared the retained first rectal CRKP isolate with the earliest retained CRKP isolate from any non-rectal anatomical site in the same patient.

For admission-relative timing, hospital day 0 was the admission date. Mutually exclusive categories were detection on days 0–2 (early-hospitalization detection), detection after day 2 with a preceding negative screen (probable within-admission screen conversion), and detection after day 2 without a preceding negative (hospital-onset detection only). Cases with insufficient admission or screening chronology were classified as indeterminate; no timing values were imputed. These categories do not establish acquisition source or transmission. Core microbiological definitions are summarized in Table 1.

## 3. Results

### 3.1. Hospital Activity, Clinical Settings, and Screening Denominators

**Figure 1:**
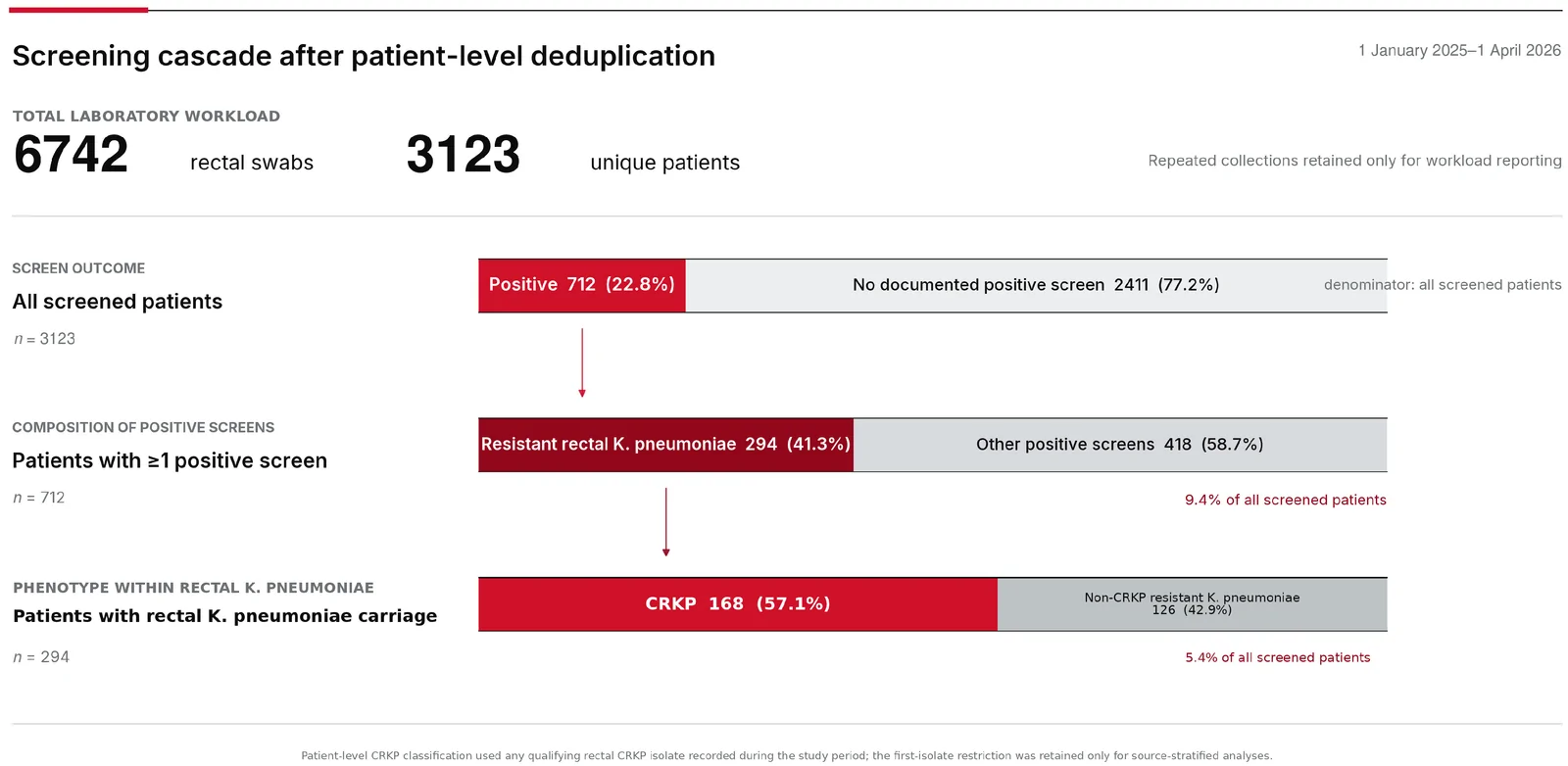
Total rectal screening workload and deduplicated patient-level prevalence. Note: The 6742 swabs include repeated collections and describe workload; prevalence used 3123 unique screened patients, with each carrier counted once. Source-stratified analyses retained the first isolate per patient, anatomical site, and microorganism. Resistance-focused screening did not estimate susceptible intestinal *K. pneumoniae* carriage.

During the study period, the hospital recorded 21,122 admission episodes. A total of 6742 rectal surveillance swabs were obtained from 3123 unique patients. This corresponded to 31.9 rectal swabs and 14.8 unique screened patients per 100 admission episodes. The total swab count describes laboratory workload and includes repeated collections; isolate-based analyses were deduplicated. Of the 6742 total rectal surveillance swabs, 1029 (15.3%) were collected in intensive-care units from 583 unique patients. A total of 3504 swabs (52.0%) were collected in surgical wards from 2013 patients, and 2209 swabs (32.8%) were collected in medical wards from 527 patients. The medical stratum included hematology and oncology. Among 3123 unique screened patients, 712 (22.8%) had at least one positive rectal result and 2411 (77.2%) had no documented positive rectal screen. Resistance-focused rectal *K. pneumoniae* carriage was documented in 294 patients (9.4% of screened patients; 41.3% of positive screens). Rectal CRKP carriage was documented in 168 patients (5.4% of screened patients; 23.6% of positive screens; 57.1% of resistance-focused rectal *K. pneumoniae* carriers). The remaining 126 carriers (42.9%) had non-CRKP resistant isolates, predominantly ESBL-annotated. Figure 1 and Table 2 summarize these denominators.

### 3.2. Rectal CRKP Carriage and Clinical-Site Detection

**Figure 2:**
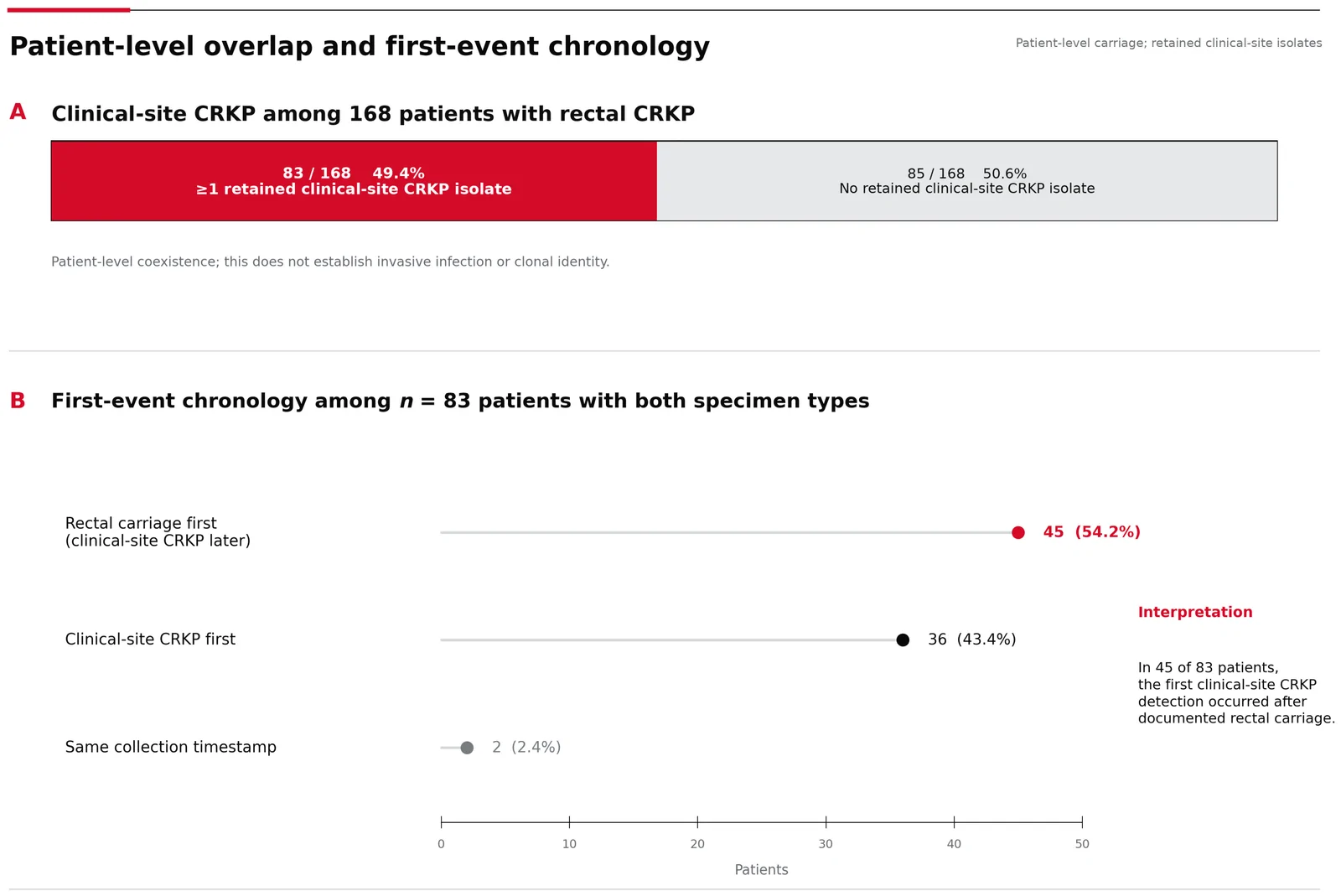
Deduplicated patient-level overlap and first-event chronology. (**A**) Same-patient overlap in the 168-patient rectal CRKP cohort; red indicates patients with at least one retained clinical-site CRKP isolate, and light gray indicates those without a retained clinical-site CRKP isolate. (**B**) First recorded collection order among the 83 patients with both specimen types; red indicates rectal carriage first, black indicates clinical-site CRKP first, and gray indicates identical collection timestamps. Recorded order does not establish progression or causality.

Among the 168 patients with patient-level rectal CRKP carriage, 83 patients (49.4%) also had at least one retained CRKP-positive clinical-site isolate from an anatomical site beyond rectal screening. The remaining 85 patients (50.6%) had rectal CRKP without a deduplicated clinical-site CRKP isolate in the available exports (Figure 2A). This overlap indicates microbiological coexistence; it does not establish infection or progression from carriage. Among the 83 patients with both retained rectal CRKP and deduplicated clinical-site CRKP isolates, rectal carriage preceded the first clinical-site CRKP detection in 45 patients (54.2%), clinical-site detection occurred first in 36 (43.4%), and the collection timestamps were identical in 2 (2.4%) (Figure 2B).

### 3.3. Anatomical Sources of Clinical-Site CRKP Detection

**Figure 3:**
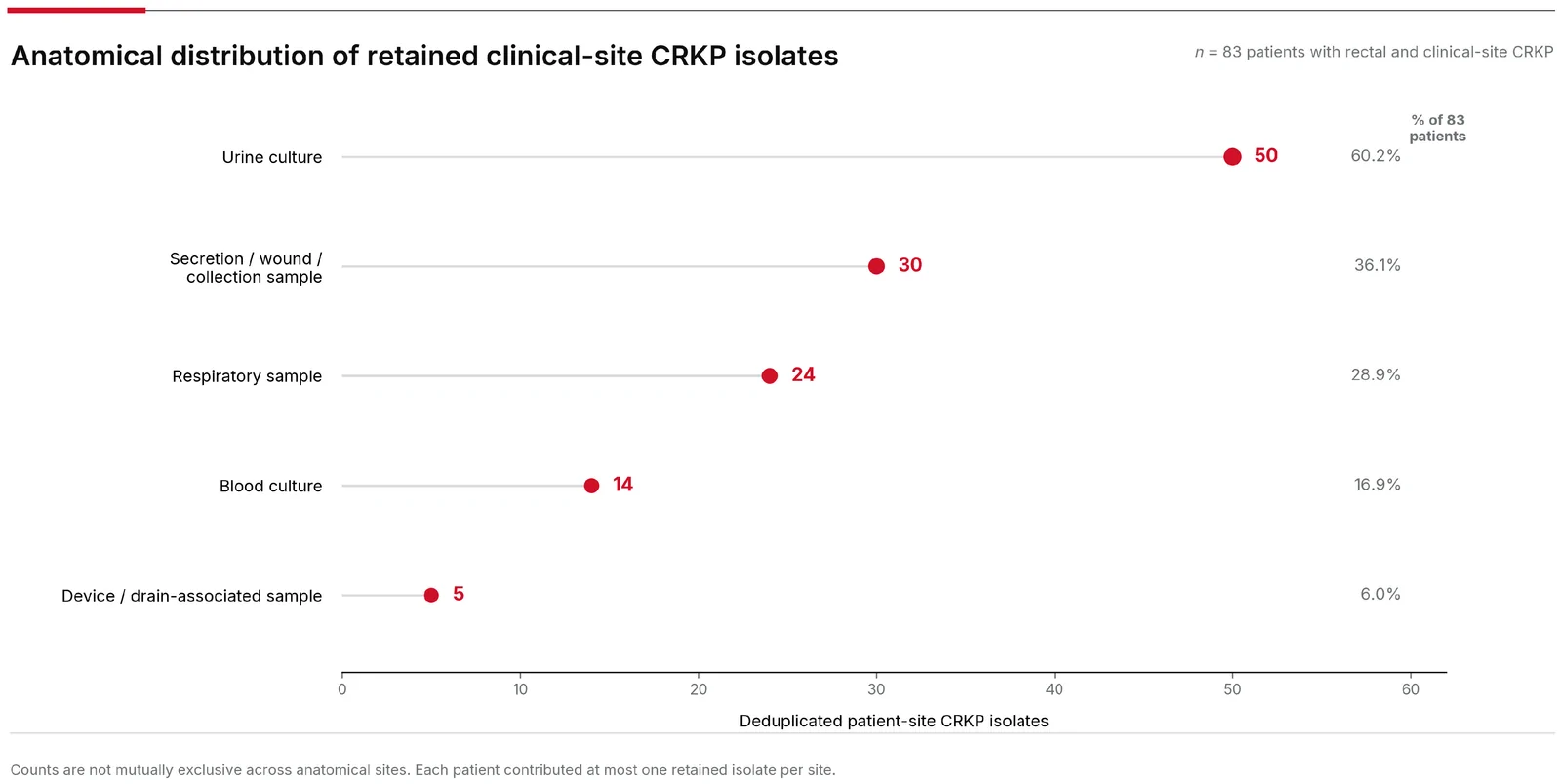
Deduplicated anatomical sources of clinical-site CRKP among patients with rectal CRKP carriage. Note: Bars show deduplicated patient–site isolates. Each patient contributed at most one *K. pneumoniae* isolate per anatomical site; counts across different sites are not mutually exclusive.

Among patients with both rectal CRKP and clinical-site CRKP detection, the most frequent deduplicated non-rectal sources were urine cultures (50 patients), secretion, wound, or collection samples (30), respiratory samples (24), blood cultures (14), and device- or drain-associated samples (5). Each patient contributed at most one *K. pneumoniae* isolate per anatomical site, although the same patient could contribute to more than one site category (Figure 3 and Supplementary Table S1).

### 3.4. Source-Stratified Phenotypic Resistance Patterns

**Figure 4:**
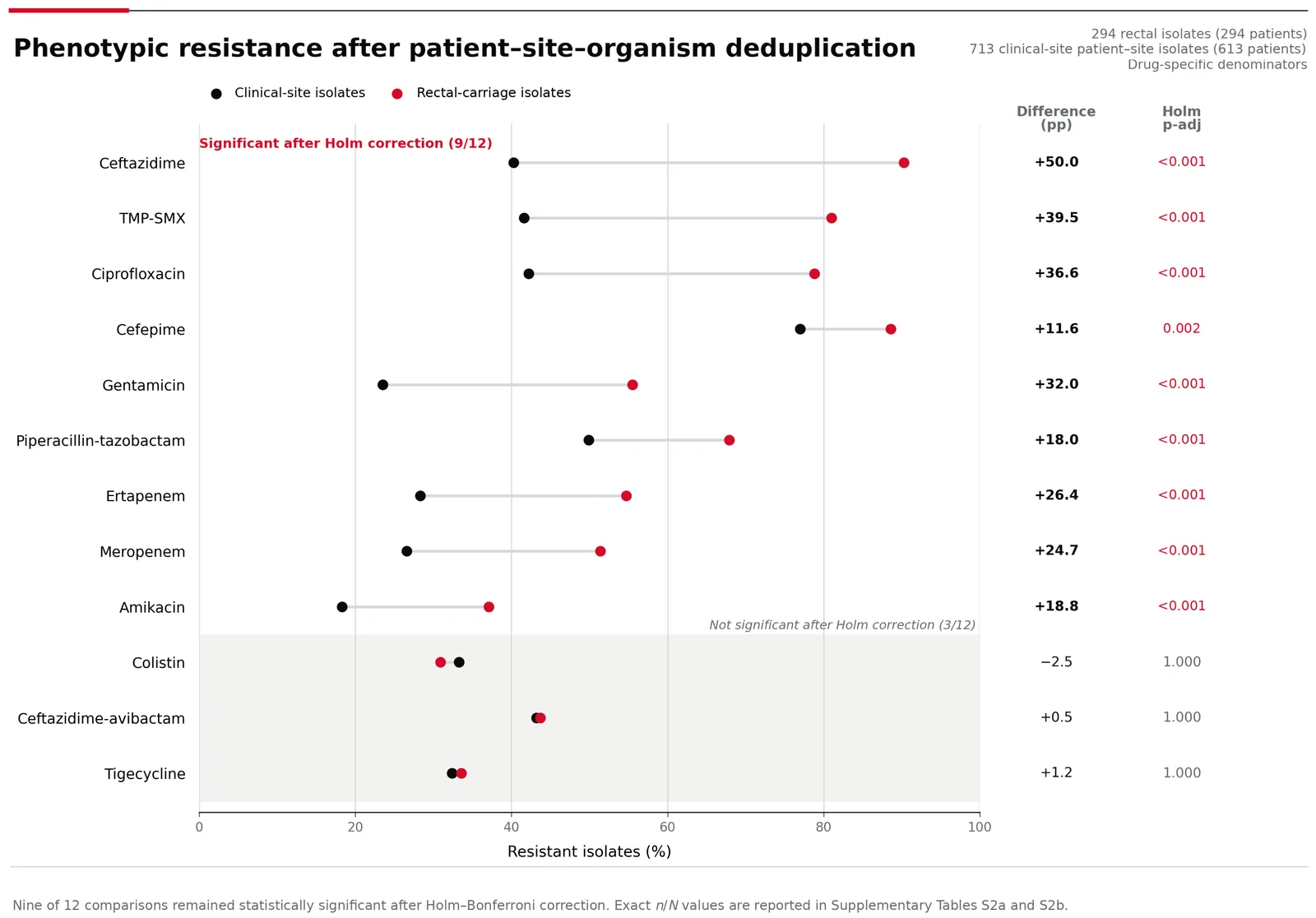
Source-stratified phenotypic resistance profile with Holm-adjusted significance after patient–site–organism deduplication. Note: Resistance proportions use drug-specific denominators. The analysis included 294 retained rectal isolates from 294 patients and 713 retained deduplicated clinical specimen isolates from 613 unique patients. Nine of 12 comparisons remained statistically significant after Holm–Bonferroni correction; exact *n*/*N* values are provided in Supplementary Table S2a,b.

Phenotypic resistance was compared between 294 first rectal isolates from 294 patients and 713 deduplicated clinical specimen isolates (one per patient and anatomical site) from 613 patients. Drug-specific denominators varied because testing was not uniform. Rectal isolates had higher resistance for most agents; 9 of 12 comparisons remained significant after Holm–Bonferroni correction (Figure 4; Supplementary Table S2a,b).

This pattern should be interpreted in relation to targeted screening and patient case mix. Source-stratified resistance differences were descriptive and should not be interpreted as source attribution or causality.

### 3.5. Descriptive Healthcare Exposure Profile of the Rectal CRKP Cohort

**Figure 5:**
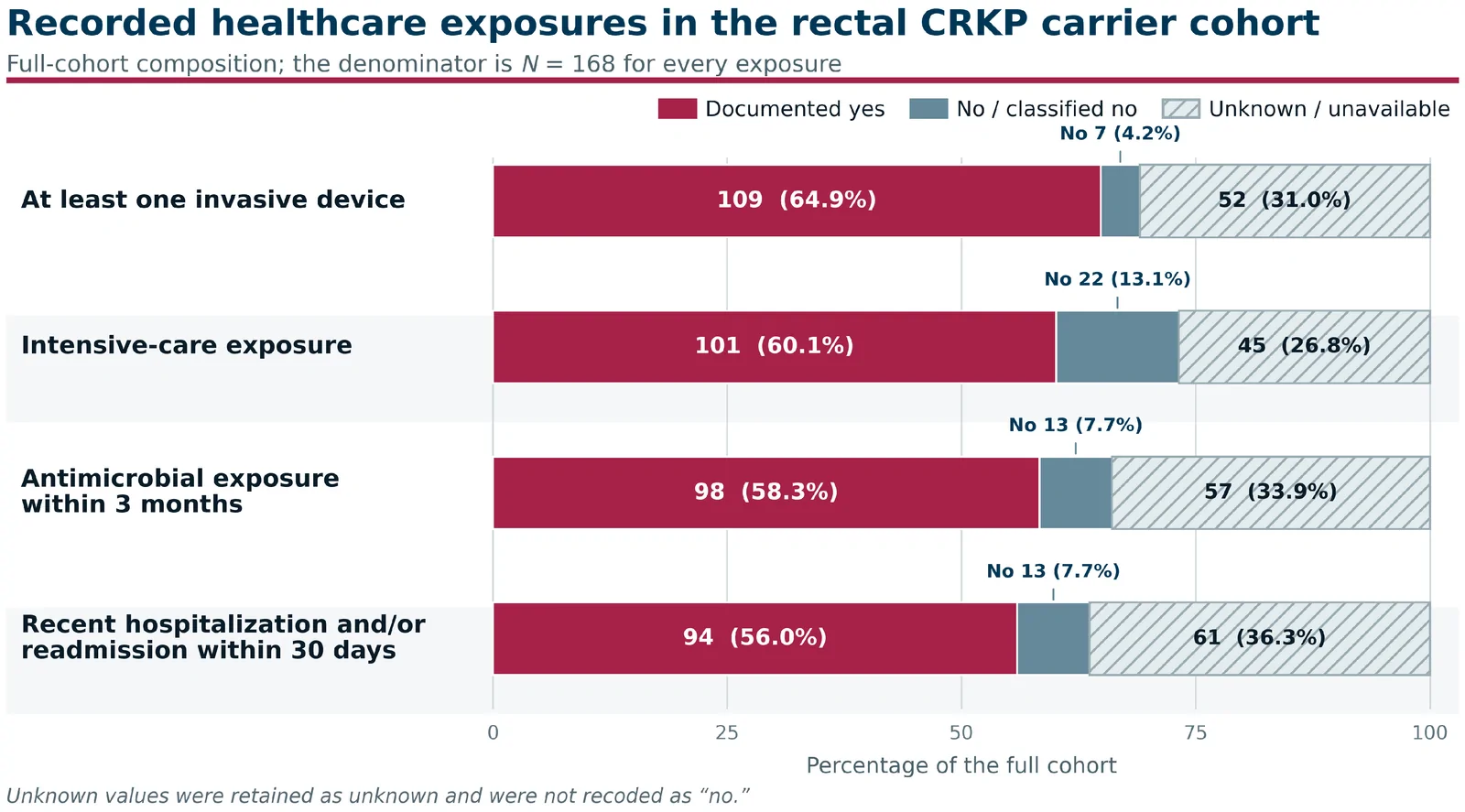
Documented healthcare exposures in the full 168-patient rectal CRKP cohort. Note: Primary percentages use *N* = 168; yes, no, and unknown sum to 168. Unknown includes 26 unlinked patients and field-specific unavailable or indeterminate values. The secondary 94/107 (87.9%) estimate applies only to patients with documented recent hospitalization/readmission status.

Clinical records were linkable for 142/168 patients (84.5%); the 26 unlinked patients remained in the *N* = 168 denominator as unknown. Recent hospitalization or readmission within 30 days, antimicrobial exposure within 3 months, exposure to at least one invasive-device, and intensive-care exposure were documented in 94 (56.0%), 98 (58.3%), 109 (64.9%), and 101 (60.1%) patients, respectively. Among the 107 patients with documented recent hospitalization/readmission status, 94 (87.9%) were exposed. These findings are descriptive and not causal risk estimates (Figure 5).

The database field for prior CRE carriage within 3 months was positive in 72/168 patients (42.9%), negative in 43/168 (25.6%), and unknown in 53/168 (31.5%). Among the 115 patients with documented status, 72 (62.6%) had prior CRE carriage. Because the underlying microbiological event date was not uniformly retained, this variable was interpreted as recorded history rather than acquisition source.

### 3.6. Episode-Level Timing of Index Rectal CRKP Detection

Among the 168 patients with rectal CRKP carriage, admission-relative classification was possible in 112 patients (66.7%). Early-hospitalization detection on hospital days 0–2 was identified in 50/168 patients (29.8%).

A documented negative-to-positive rectal screening sequence after hospital day 2 supported classification as probable within-admission screen conversion in 30/168 patients (17.9%). Hospital-onset detection after day 2 without a documented prior negative occurred in 32/168 patients (19.0%) and was retained as detection only. Classification remained indeterminate in 56/168 patients (33.3%). Neither early nor hospital-onset detection alone established community origin or within-admission acquisition.

## 4. Discussion

This study provides hospital-specific estimates of screen-detected rectal CRKP carriage and describes same-patient clinical-site detection, collection order, healthcare exposures, prior CRE carriage, and admission-relative timing in a selected screened population. Same-patient overlap indicates microbiological coexistence but does not establish infection, progression, acquisition, transmission, or clonal identity. Healthcare exposures and prior carriage were frequently documented, but incomplete time anchoring and the absence of a comparator group precluded inference regarding acquisition source or independent risk factors. Admission-relative categories described detection timing rather than importation or within-admission acquisition. Source-stratified resistance differences were likewise descriptive because screening was targeted and test availability varied by agent.

### 4.1. Limitations

This retrospective single-center study used laboratory exports from a non-universally screened population; prevalence therefore applies only to screened patients. Resistance-focused screening did not estimate total intestinal *K. pneumoniae* carriage. Admission-relative timing was classifiable for 112/168 patients, while 56 were indeterminate; prior-carriage status lacked a uniform date and was unknown in 53/168. Intermittent risk-based screening and incomplete chronology limited interpretation of negative-to-positive sequences and hospital-onset detections. The study therefore could not distinguish community, long-term-care, environmental, or within-hospital acquisition or assess acquisition during a prior hospitalization, isolation failure, or equipment contamination.

Clinical infection was not systematically adjudicated; accordingly, clinical-site CRKP recovery could not be classified uniformly as infection or colonization.

Molecular typing was not performed for all isolates. Patient-level overlap therefore indicates the same species-resistance phenotype, not proven clonal relatedness. Paired molecular typing, ideally WGS, would be needed to assess genetic relatedness; inference of transmission would additionally require epidemiological correlation [21].

Antimicrobial susceptibility testing used drug-specific denominators, and not all agents were tested in all isolates. The first-isolate design excluded later same-site isolates and therefore did not capture subsequent phenotypic changes.

Carbapenemase-gene testing and MLST were not uniformly available. The absence of a comparator cohort and time-anchored covariates precluded risk-factor modeling; related institutional findings have been reported separately [22].

### 4.2. Future Directions

Prospective studies should combine admission and serial rectal screening with time-anchored clinical data, infection adjudication, and molecular typing to distinguish detection timing, assess strain relatedness and transmission [21], and identify independent risk factors [23].

## 5. Conclusions

Rectal CRKP carriage was frequent and commonly associated with same-patient CRKP detection at non-rectal clinical sites, indicating a substantial hospital-associated burden. These findings support incorporating documented CRKP carriage into clinical decision-making: in critically ill patients with sepsis or septic shock, known carriage status should inform the choice of empirical antimicrobial therapy toward CRKP-active coverage, with prompt de-escalation once microbiological findings and clinical response allow. Given the descriptive design and absence of comparator group and molecular typing, prospective molecular studies are needed to clarify acquisition and transmission pathways.

## Figures and Tables

**Table 1:** Operational definitions used for denominator-based interpretation.

Term	Operational Definition and Clinical Interpretation
Screened population	Unique patients with at least one rectal surveillance swab, regardless of result. This is the denominator for patient-level prevalence.
Positive rectal screening	At least one positive rectal surveillance swab for any organism. This describes screening positivity, not species-specific prevalence.
Resistance-focused rectal *K. pneumoniae* carriage	At least one qualifying rectal *K. pneumoniae* isolate during the study period; each patient counted once. Prevalence used all screened patients.
Rectal CRKP carriage	At least one qualifying rectal *K. pneumoniae* isolate resistant to at least one of ertapenem, imipenem, or meropenem during the study period; interpreted as gastrointestinal carriage, not active infection.
Clinical-site CRKP detection	Retained first K. pneumoniae isolate from a non-rectal anatomical site that was resistant to at least one of ertapenem, imipenem, or meropenem. This is microbiological detection, not automatically invasive infection.
Patient-level overlap	The same patient had retained rectal CRKP and at least one retained clinical-site CRKP isolate. This indicates the same species-resistance phenotype at patient level; strain relatedness requires molecular typing.
Deduplicated isolate	Earliest chronologically documented isolate for a given patient, anatomical site category, and microorganism. Repeated isolates of the same microorganism from the same site were excluded.

Note. Patient-level prevalence used 3123 unique screened patients, with each carrier counted once. Source-stratified analyses retained the earliest isolate per patient, anatomical site category, and microorganism. Because screening was resistance-focused, a negative screen did not exclude susceptible *K. pneumoniae* carriage.

**Table 2:** Total workload and deduplicated patient-level screening estimates.

Measure	Count	Patient-Level Denominator/Interpretation
Total rectal surveillance swabs	6742	Workload only; repeated collections retained
Unique screened patients	3123	3123/3123 (100.0%)
At least one positive rectal screen	712	712/3123 (22.8%)
No documented positive rectal screen	2411	2411/3123 (77.2%)
Patient-level rectal *K. pneumoniae* carriage	294	294/3123 (9.4%)
Patient-level rectal CRKP carriage	168	168/3123 (5.4%)

## Data Availability

The anonymized aggregate data supporting this analysis are available from the corresponding author upon reasonable request and subject to institutional governance requirements.

## Supplementary Materials

The following supporting information can be downloaded at: https://www.mdpi.com/article/10.3390/antibiotics15080764/s1, Table S1: Deduplicated anatomical sources of clinical-site CRKP among patients with retained rectal CRKP; Table S2a: Deduplicated source-stratified phenotypic antimicrobial resistance comparisons that remained statistically significant after Holm–Bonferroni correction; Table S2b: Deduplicated source-stratified phenotypic resistance comparisons without statistical significance after Holm–Bonferroni correction.

## Abbreviations

AST, antimicrobial susceptibility testing; CRE, carbapenem-resistant Enterobacterales; CRKP, carbapenem-resistant *K. pneumoniae*; ESBL, extended-spectrum beta-lactamase; EUCAST, European Committee on Antimicrobial Susceptibility Testing; MIC, minimum inhibitory concentration; *p*-adj, adjusted *p*-value; pp, percentage points; TMP-SMX, trimethoprim-sulfamethoxazole; WGS, whole-genome sequencing.
