# Supplementary material for: Rectal Surveillance for Carbapenem-Resistant Klebsiella pneumoniae: Prevalence and Clinical Relevance in a Multidisciplinary Tertiary-Care Hospital
Source: Antibiotics (Basel). 2026 Aug 10;15(8):764. doi: 10.3390/antibiotics15080764 (PMC13509979; doi:10.3390/antibiotics15080764)
Supplement: Supplementary file 1 [file antibiotics-15-00764-s001.zip › antibiotics-4454345-supplementary.pdf]

Supplementary Tables

Supplementary Table S1. Deduplicated anatomical sources of clinical-site CRKP among patients with retained rectal CRKP.

Specimen type	Deduplicated isolates	Unique patients
Urine culture	50	50
Secretion / wound / collection sample	30	30
Respiratory sample	24	24
Blood culture	14	14
Device / drain-associated sample	5	5

Each patient contributed at most one K. pneumoniae isolate per anatomical site; therefore, deduplicated isolate counts equal unique-patient counts within each row. Patients could contribute across more than one site category.

Supplementary Table S2a. Deduplicated source-stratified phenotypic antimicrobial resistance comparisons that remained statistically significant after Holm-Bonferroni correction.

Antimicrobial agent	Rectal surveillance isolates, R n/N (%)	Clinical-specimen isolates R n/N (%)	Difference (pp)	p-adj
**Ceftazidime**	252/279 (90.3%)	278/689 (40.3%)	**+50.0**	**< .001**
**TMP-SMX**	218/269 (81.0%)	279/671 (41.6%)	**+39.5**	**< .001**
**Ciprofloxacin**	231/293 (78.8%)	300/711 (42.2%)	**+36.6**	**< .001**
**Cefepime**	210/237 (88.6%)	218/283 (77.0%)	**+11.6**	**.002**
**Gentamicin**	146/263 (55.5%)	154/655 (23.5%)	**+32.0**	**< .001**
**Piperacillin-tazobactam**	199/293 (67.9%)	247/495 (49.9%)	**+18.0**	**< .001**
**Ertapenem**	152/278 (54.7%)	193/682 (28.3%)	**+26.4**	**< .001**
**Meropenem**	151/294 (51.4%)	189/710 (26.6%)	**+24.7**	**< .001**
**Amikacin**	93/251 (37.1%)	120/657 (18.3%)	**+18.8**	**< .001**

Supplementary Table S2b. Deduplicated source-stratified phenotypic resistance comparisons without statistical significance after Holm-Bonferroni correction.

Antimicrobial agent	Rectal surveillance isolates, R n/N (%)	Clinical-specimen isolates R n/N (%)	Difference (pp)	p-adj
**Colistin**	63/204 (30.9%)	85/255 (33.3%)	**−2.5**	**1.000**
**Ceftazidime-avibactam**	108/247 (43.7%)	114/264 (43.2%)	**+0.5**	**1.000**
**Tigecycline**	46/137 (33.6%)	56/173 (32.4%)	**+1.2**	**1.000**

R, resistant; pp, percentage points; p-adj, Holm-Bonferroni-adjusted p-value. One isolate was retained per patient, anatomical site, and microorganism. Denominators vary by antimicrobial agent because testing was not uniform.
